# A Review on N-Doped Biochar for Oxidative Degradation of Organic Contaminants in Wastewater by Persulfate Activation

**DOI:** 10.3390/ijerph192214805

**Published:** 2022-11-10

**Authors:** Yaxuan Gao, Wenran Gao, Haonan Zhu, Haoran Chen, Shanshan Yan, Ming Zhao, Hongqi Sun, Junjie Zhang, Shu Zhang

**Affiliations:** 1 Co-Innovation Center of Efficient Processing and Utilization of Forest Resources, Joint International Research Laboratory of Biomass Energy and Materials, College of Materials Science and Engineering, Nanjing Forestry University, Nanjing 210037, China; 2 School of Engineering, Edith Cowan University, Joondalup, WA 6027, Australia; 3 State Key Laboratory of Organic Electronics and Information Displays, Institute of Advanced Materials, School of Material Science and Engineering, Nanjing University of Posts and Telecommunications, Nanjing 210023, China

**Keywords:** N-doped biochar, persulfate, advanced oxidation process, N configuration, wastewater

## Abstract

The Persulfate-based advanced oxidation process is the most efficient and commonly used technology to remove organic contaminants in wastewater. Due to the large surface area, unique electronic properties, abundant N functional groups, cost-effectiveness, and environmental friendliness, N-doped biochars (NBCs) are widely used as catalysts for persulfate activation. This review focuses on the NBC for oxidative degradation of organics-contaminated wastewater. Firstly, the preparation and modification methods of NBCs were reviewed. Then the catalytic performance of NBCs and modified NBCs on the oxidation degradation of organic contaminants were discussed with an emphasis on the degradation mechanism. We further summarized the detection technologies of activation mechanisms and the structures of NBCs affecting the PS activation, followed by the specific role of the N configuration of the NBC on its catalytic capacity. Finally, several challenges in the treatment of organics-contaminated wastewater by a persulfate-based advanced oxidation process were put forward and the recommendations for future research were proposed for further understanding of the advanced oxidation process activated by the NBC.

## 1. Introduction

With the rapid development of urbanization and industrialization in modern societies, environmental crises have drawn the world’s attention towards a sustainable future [1,2]. Over the past decades, the deterioration of water resources has always been a serious problem. Therefore, a variety of technologies have been developed to remedy various organic pollutants, including antibiotics, dyes, phenols, and pesticides, in water matrices [3,4,5]. For example, physical methods such as adsorption and flocculation can easily remove harmful substances from water by transferring them from one phase to another, but essentially they cannot remove organic matter completely [6]. Biological methods include the aerobic-activated sludge method and the sludge anaerobic digestion biodegradation method which can also remove organic contaminants via microorganisms; however, because microorganisms have selectivity to the degradation of pollutants, they cannot completely degrade and mineralize the pollutant molecules thus making the effluent unable to meet the water quality requirements [7]. Therefore, it is urgent to develop an efficient and environmentally friendly technology to control water pollution. The chemical method is an effective alternative. The advanced oxidation processes (AOPs) with the generation of free radicals as the core is a new technology for the treatment of organic pollutants in water in recent years [8,9,10]. For chemically oxidization-based AOPs, the free radicals produced by various activated peroxides (e.g., ozone, persulfates, and hydrogen peroxide, etc.), such as hydroxyl radicals (·OH), sulfate radicals (SO_4_^·−^), and superoxide ion radicals (O_2_^·−^), are generally considered as the major species of reactive oxygen species (ROSs) that cause organic degradation. In these cases, hydroxyl radicals (·OH) produced in the Fenton or Fenton-like processes always have disadvantages such as pH restriction (pH = 3–4), low catalytic efficiency of Fe^2+^, and high quenching of ·OH reactions [11]. Compared with Fenton oxidation, persulfate-based AOPs (PS-AOP) have the advantages in terms of higher redox potential, wider pH range, and a longer half-life period [8,12].

**Figure 1:**
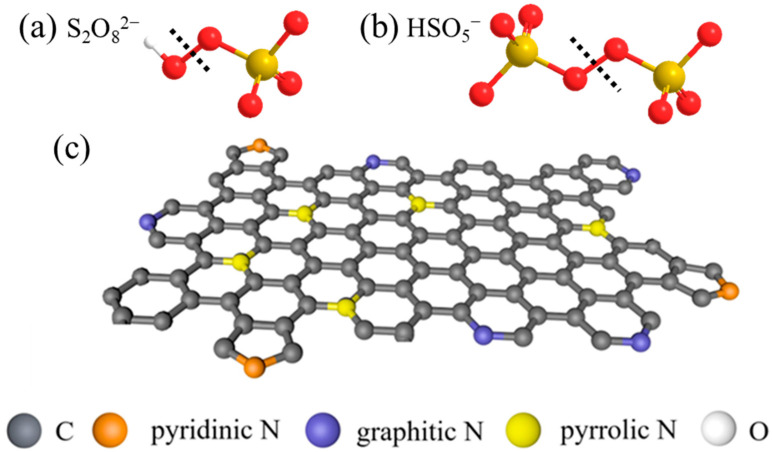
The structure of (**a**) PDS and (**b**) PMS, and (**c**) the structure of different N species [39], copyright 2021, Elsevier.

In general, persulfates (PSs), such as peroxymonosulfate (PMS) and peroxydisulfate (PDS) shown in Figure 1a,b, has a low oxidative potential (2.01 V and 1.82 V for PDS and PMS, respectively) for organic decomposition [13,14,15]. Therefore, the PS-AOP relies on the reactive species produced by PS activation to degrade pollutants, such as the generated SO_4_**^·^**^−^ has a pair of arc pair electrons which makes it have a high oxidation potential (2.5–3.1 V) [16]. The type of produced reactive species depends on the activation mechanism of the PS, including radical and nonradical pathways. The radical pathway produces a highly reactive SO_4_**^·^**^−^ and ·OH from PDS/PMS through O-O bond cleavage. The nonradical pathway produces singlet oxygen (^1^O_2_) through nucleophilic addition of PMS, or forms the surface activate complex by binding PMS/PDS onto the catalyst surface (electron transfer) [17].

One of the key factors of the PS-AOP is the catalyst which can determine the type of PS activation pathway and the efficiency of the pollutants degradation. In PS-AOP, catalysts can be roughly divided into two types: homogeneous catalysts dominated by transition-metal ions [18], and heterogeneous catalysts including zero-valent metals [19], metal oxides [20], and carbon-based materials [21,22]. Homogeneous catalysts possesse better reaction efficiency but causes a secondary pollution resulting from metal ion leaching. Therefore, a heterogeneous catalyst is more commonly used in PS-AOP. Recently, carbon-based materials have been used as a new type of green catalyst due to their large surface areas, unique electronic properties, sp^2^-hybridized carbon (Sp^2^-C) configuration, and non-secondary pollution [23,24,25,26,27]. Because commonly used carbon precursors in carbon catalysts (such as fullerenes, carbon nanotubes, and graphene oxide) are expensive, it is difficult to use them on a large scale and they are hazardous to the environment. Therefore, low cost and an environmentally friendly biomass that can be applied on a large scale are promising materials for the preparation of carbon catalysts [28,29,30].

Recently, biochars derived from biomass waste such as spend coffee grounds, sludge, rice straw, and corncobs have drawn increasing attention as a potential carbon-based catalyst for pollutant removal [31,32,33,34]. As a carbon-based catalyst, biochars are usually produced by the slow pyrolysis of a biomass in anoxic or anaerobic environments; however, the primary biochar has limited catalytic activity due to the disordered structure of amorphous carbon, which cannot effectively realize directional charge transfers. As an effective method to induce a Sp^2^-C skeleton to offer more active sites and to regulate its electronic properties, heteroatom doping, especially N-doping, has attracted extensive attention [35,36,37,38]. High-electron cluster density is required to start the catalytic process. Under certain electron environments, electron reconfiguration can be caused by accelerated electron transfer and heteroatomic doping, which is a theoretically feasible approach for PS-activation-state formation and active species generation. Introducing a heteroatom into the carbon lattice increases the degree of charge delocalization, thus breaking the inertia of the Sp^2^-C network structure.

As shown in Figure 1c, for the N-doped biochar (NBC), pyridinic N, pyrrolic N, and graphitic N are the three main N-bonding configurations in the carbon networks structure [40]. Pyrrolic N with sp^2^ hybridization exists in the six-membered ring bonding of two C atoms at the edges of NBC, providing one p electron for the π-system. Pyridinic N with sp^2^ hybridization can exist in a five-membered ring, providing two p electrons to the aromatic system [41]. Graphitic N generally refers to the bonding of sp^2^-hybridized N atoms with three sp^2^-C atoms [42,43]. However, there is a lack of review on the role of different N configuration in PS-AOPs.

Therefore, in order to reveal the relationship between the N configuration and the catalytic efficiency/mechanism of NBC in PS-AOP, we believe that it is timely to conduct a comprehensive review of the articles and progress of NBC, especially with respect to its synthesis process, applications, performance, and feasibility. This review includes four main parts: (1) Overview of NBC preparation and modification methods; (2) Catalytic ability of NBCs and modified NBCs on PS activation to degrade organic contaminants and the detection technologies for PS activation mechanism; (3) How the structure of NBCs are affecting the PS activation and the role of N configuration; (4) Recommendations for future work.

## 2. Preparation and Modification of NBC

The preparation methods of NBCs can be mainly divided into two types: in situ method and the post-treatment method. The in situ method uses naturally N-rich biomasses directly as N sources for N-doping, such as sludge, spirulins residue, lotus leaf, bean dreg, etc. The post-treatment method uses N-based chemicals as the N dopant including urea, melamine, thiourea, NH_4_OH, NH_4_NO_3_, etc. In addition, the catalytic performance of NBCs can also be improved by co-doping with other elements (i.e., metal element and non-metal element). The specific NBC synthesis and modification is described below.

### 2.1. Preparation Methods of NBC

N-doping is considered to be an effective method to enhance the catalytic performance of inert carbonaceous materials by tuning the spin density and charge distribution [31,44,45,46,47]. As shown in Table 1, we summarized the recent NBC materials for PS-AOP. We found that aquatic plants such as lotus leaves, spirulina residue, and water hyacinth are always used as biomass feedstock for the in situ method. The reason may be related to the fact that aquatic plants can absorb inorganic N from water sludge. Liu et al. [48] reported that water hyacinth can accumulate a considerable amount of the inorganic N in domestic sewage, while the free-floating perennial plant contains about 30% protein. Since these aquatic plants can obtain N from sludge, sludge is also a good natural N source. Yin et al. [49] reported the N content of the N-enriched biochar prepared from sludge reached up to 4.94%. The content of graphitic N of biochar prepared from sludge is particularly prominent, which proves to be a favorable N configuration of PS-AOP [50]. In addition, external N sources can be added to the sludge to prepare NBCs. Yin also reported that through the co-pyrolysis of sludge and urea, the N content was increased from 4.94% to 11.16%, and the corresponding catalytic efficiency was also greatly increased.

In addition to sludge, bamboo biomass is also a suitable feedstock with the addition of external N dopants. Zhang et al. [51] reported that N content of maso bamboo biochar increased from 0.45% to 5.04% after N-doping. There are also some biomass materials that are not suitable for the preparation of NBCs due to their own characteristics. Oh et al. [52] prepared NBCs by the co-pyrolysis of different biomass materials with urea, and found that the structural characteristics of the biomass materials have an important influence on the degree of N-doping. Biomass with high inorganic content (e.g., banana peels, rice straw, etc.) may not suitable for the preparation of NBCs, because the high content of inorganic salts reduces the C content which is unfavorable for N-doping [53]. In addition, the presence of veins in the biomass (e.g., dry leaf) leads to a low specific surface area (SSA) of NBCs, because the chemical doping is more difficult for this structure [52].

Xu et al. [54] investigated how the different organic N-containing compounds including urea, melamine, thiourea, and dicyandiamide affect the catalytic activity of the NBC. Mian et al. [50] prepared NBC from widespread sewage sludge with inorganic N dopant (i.e., NH_4_OH), and the NBC deposited a large amount of inorganic salts on its surface which is not favorable for PS-AOP. Therefore, urea is currently the main N dopant for the preparation of NBCs for PS-AOP. However, the dominant N configuration of NBCs prepared by urea is different for different biomasses. Therefore, the precise synthesis of NBCs is still challenging.

**Table 1:** Preparation of N-doped biochar from different biomass by pyrolysis.

Method	Biomass	N-Dopant	Temperature (°C)	N Content of Biochar (%)	SSA (m^2^g^−1^)	Ref.
In situ	Spirulins residue		400–900	0.77–3.61	67–117.9	[55]
	Water hyacinth		600–800	2.8–5.02	700.6–1199.3	[48]
	Candida utilis		700	3.69–5.91	3.8–47.1	[56]
	Sludge		700	4.94	-	[49]
	Lotus leaf		700–900	1.58–3.43	118.93–360.49	[57]
	Bean dreg		400–900	1.27–3.23	31.6–3194.9	[58]
	Passion fruit shell		900	1.43	536.55	[59]
Post- treatment	Dry leaf	Urea	1000	1.0	118	[52]
	Spend coffee ground	Urea	1000	2.1	439	
	Banana peel	Urea	1000	1.1	450	
	Orange peel	Urea	1000	1.0	238	
	Saw dust	Urea	1000	0.3	423	
	Corncob	Urea	700	11.36	-	[49]
	Sludge	Urea	700	11.16	-	
	Wood residue	Urea	800	12.1	588	[60]
	Rice straw	Urea	1000	4.39	158.3	[61]
	Sludge	Urea	700	0.39	161.004	[62,63]
	Rice straw	Urea	700–900	0.12–18.35	333.7–514.3	[64]
	Sludge	Urea	500–800	12.141–24.968	241.85–370.54	[65]
	Spend coffee powder	Urea	500–1000	16.6–25.7	23.3–438.8	[33]
	Moso bamboo	Urea	700	5.04	250.31	[51,66]
	Sewage sludge	Urea	300–900	0.081–0.384	14.4–36.5	[62,67]
	Corncob	Urea	700	10.43	4.98	[31]
	Pine-wood	2-methylimidazole	800	2.06	1398	[64,66]
	Straw	Thiourea	700–900	2.21–4.35	417.24–570.74	[68]
	Sludge	NH_4_OH	600	3.9	50.6	[50,51]
	Reed	NH_4_NO_3_	400–900	1.76–8.11	71.5–498.7	[63,67]
	Sawdust	Dicyandiamide	800	19.53	174.45	[50,54]

### 2.2. Modification of NBC

Co-doping with other atoms is considered as a modification method to enhance the catalytic performance of NBCs. The co-doped elements are mainly divided into non-metallic elements (e.g., S, B) and metal elements (e.g., Fe, Cu, Co).

Boron is one of the main non-metallic elements co-doped in NBCs because its atomic size is similar to that of C atom, and it is easy to incorporate into the C grid (usually ‘B-C’ and ‘B-O’ are incorporated into the middle of C grid). B-doping induces a shift in the conduction band of the Fermi level, which can improve the surface electron transfer of carbon materials [69]. B-doping also results in the grafting of oxygen to the carbon surface, improving the electron plane and chemical stability of carbon. Due to the high stability of the boron group, the B-doped biochar has better long-term durability than the N-doped carbon [70].

As a non-metallic element, the incorporation of S atom into NBCs for PS-AOP has attracted extensive attention. S atoms are generally incorporated into NBCs in the form of “C-S-C”. Since its electronegativity is higher than that of C atoms, the incorporation of S atoms into the C matrix can improve charge transfer capacity [71,72]. At present, there are two main preparation methods for S-doped NBCs (S-NBC). One is one-step pyrolysis that directly co-pyrolysis the biomass and N/S-rich precursors (such as thiourea, tert-butanol). The other is two-step pyrolysis that co-pyrolysis the N-containing precursor and biomass first and then co-pyrolysis with S-containing precursor (e.g., thiophene). The catalytic effect of S-NBC prepared by one-step pyrolysis is not as good as that of NBC [51,61] prepared under the same conditions, while the catalytic effect of S-NBC prepared by two-step pyrolysis (sequential impregnation) method is stronger [73]. This shows that the properties of S-NBC manufactured by different preparation methods are different. Therefore, further research on the synthesis and catalytic mechanism of non-metallic element-co-doped NBC is needed, which will be further discussed in Section 3.2.

Metal atoms can also be incorporated into NBCs to enhance the PS-AOP catalytic degradation ability [74,75]. Doping with N and Cu (Cu-NBC) is an effective way to prepare high-efficiency biochar-based catalysts. The prepared Cu-NBC had massive carbon structure and urchin-like structure of Cu, and the Cu were the main active substances. This material has advantages including low consumption of PS, strong pollutant degradation efficiency, and suitability for a wide range of pH conditions [75]. Similarly, co-doping Co into NBC can synergistically enhance the catalytic activity of the catalyst for PMS/PDS activation. The valence state transition of the divalent and trivalent Co ions has a significant impact on the activation mechanism [76].

**Figure 2:**
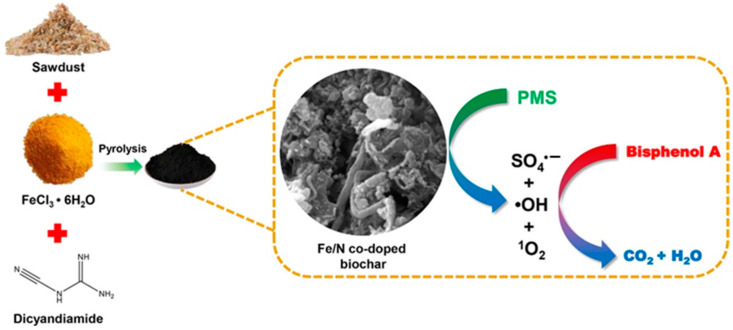
Schematic illustration of the preparation of Fe-NBC [78], copyright 2020, Elsevier.

In recent years, many researchers have demonstrated that Fe and N co-doped biochars (Fe-NBCs) have high catalytic performance in PS-AOP [74,77,78,79,80]. As shown in Figure 2, Xu et al. [78] used dicyandiamide as the N source and FeCl_3_ as the iron source, respectively, and then heated, stirred, and dried with sawdust in water to obtain Fe-NBC. The prepared Fe-NBC has high SSA and abundant defects. Fe and the synergistic effect exhibited by N after co-doping endows it with superior catalytic ability in PS-AOP (removal efficiency =97%). Woody biomasses such as corn stover [79,81] and rice husk [77] can be used to prepare Fe-NBC using a impregnation-pyrolysis method. For sludge biomass, the source of sludge needs to be paid attention. Wu et al. [82] studied sludge from different sources and found that the municipal sewage sludge is rich in metals and other insoluble substances. In the process of preparing Fe-NBCs from municipal sewage sludge, Fu et al. [74] used ethylene diamine tetraacetic acid (EDTA)-citric acid for pretreatment to recover the heavy metals in the sludge. Therefore, for the sludge extracted from urban domestic sewage, it is necessary to pay attention to the risk of metal overflow if mixed with metal elements. Doping metal atoms can effectively enhance the catalytic activity of NBC, but precise control of metal incorporation is a major challenge in synthesis. In addition, the synergistic mechanism of different metals should be extensively studied.

## 3. Catalytic Performance of NBC on Persulfate Activation and the Activation Mechanism

### 3.1. NBC for PS-AOP

The characteristics of a NBC are high SSA, a certain degree of graphitization and defects, abundant N functional groups, etc. Table 2 summarizes the previous work using NBC catalysts to activate PDS/PMS to degrade organic pollutants.

For the current activation mechanism of NBCs involved in PS-AOP, the free-radical pathway degradation mechanism represented by SO_4_^·−^ and ·OH is relatively simple. NBCs can act as electron donors and activate PS by electron transfer through the cleavage of O-O bond and thereby produce SO_4_^·−^ and ·OH as follow (Equations (1) and (2)).

(1) $$HSO_{5}^{-}+e^{-}\to SO_{4}^{•-}+\cdot OH$$

(2) $$S_{2}O_{8}^{2-}+e^{-}\to 2SO_{4}^{•-}$$

Then pollutants (electron donor) can be oxidized into CO_2_ and H_2_O by SO_4_**^·^**^−^ and ·OH (electron acceptor) as follows (Equations (3) and (4)).

(3) $$Pollutants+SO_{4}^{•-}\to intermediates\to SO_{4}^{2-}+H_{2}O+CO_{2}$$

(4) $$Pollutants+\cdot OH\to intermediates\to H_{2}O+CO_{2}$$

However, the free-radical pathway usually only plays an auxiliary role in the NBC/PS system, and the reason may be that the incorporation of N enhances the non-radical pathway [83]. Pei et al. [62] prepared NBCs (i.e., urea-doped sludge biochar) to activate PDS for the degradation of sulfadiazine. It demonstrated that the doping of N atoms positively charged the adjacent C atoms, thereby allowing the electrons to interact with S_2_O_8_^2−^ by direct transfer and generate ^1^O_2_ via non-radical pathways (Equation (5)) [84].

(5) NSBC/SO3−O−O−SO32−→NSBC/O21+2SO42−

In addition, the C=O functional group formed during the pyrolysis of NBC can also generate ^1^O_2_ in a similar manner, see Equations (6)–(8) [12].

(6) $$NSBC=O+S_{2}O_{8}^{2-}+OH^{-}\to NSBC-OH/SO_{3}-O-O^{-}+SO_{4}^{2-}$$

(7) $$NSBC-OH/SO_{3}-O-O^{-}+OH^{-}\to NSBC-O^{-}/SO_{3}-O-O^{-}+H_{2}O$$

(8) NSBC−O−/SO3−O−O−+S2O82−+2OH−→NSBC=O+3SO42−+O21+H2O

**Table 2:** List of N-doped biochar as catalysts for PS-AOP.

Biomass	Oxidant	Catalysts	Pollutant	Reaction Conditions	Removal Efficiency (%)	Rate Constant (min^−1^)	Active Sites	Activation Mechanism	Ref.
Raw silk	PMS	PGBF-N-900	Tetracycline	T = 25 °C, pH = 7, Catalyst = 0.1 g/L, [PMS] = 1 mM, [TC] = 20 mg/L	96.5	0.0206	C=O, Graphitic N, Defect sites	SO_4_^·−^, ·OH, ^1^O_2_, Electron transfer	[85]
Corncob	PDS	NBC3	Sulfadiazine	T = 25 °C, pH = 7, Catalyst = 1.0 g/L, [PDS] = 1 mM, [SDZ] = 10 μM	96.5	0.0748	Pyridinic N, Pyrrolic N, C-N atoms	Electron transfer	[31]
Candida utilis	PMS	NCS-6	Bisphenol A	T = 25 °C, pH = 7, Catalyst = 0.4 g/L, [PMS]= 0.4 g/L, [TC] = 20 mg/L	100	1.36	Sp^2^-C, Defect sites, Graphitic N, Pyridinic N	SO_4_^·−^, ·OH, ^1^O_2_, Electron transfer	[56]
Sludge	PDS	NSBC-700	Sulfadiazine	pH = 3.1, Catalyst = 1.0 g/L, [PDS] = 600 mg/L, [SD] = 20 mg/L	97	-	C (adjacent to N atom), C=O, Pyridinic N	Surface-bound radical, ^1^O_2_	[62]
Sludge	PMS	NC-700	Methylene blue	T = 25 °C, Catalyst = 0.3 g/L, [PMS] = 0.4g/L, [MB] = 50 mg/L	93.2	0.3009	Graphitic N, C=O	^1^O_2_, SO_4_^·−^, ·OH	[65]
Pinewood	PMS	NKBC800	Ciprofloxacin	T = 25 °C, Catalyst = 0.2 g/L, [PMS]= 3 mg/L, [CIP] = 50 mg/L	87	0.053	C=O, Pyridinic N, Sp^2^-C	SO_4_^·−^, ·OH, ^1^O_2_, Electron transfer	[66]
Spirulina residue	PDS	SDBC900	Sulfamethoxazole	T = 25 °C, Catalyst = 0.5 g/L, [PDS]= 6 mM, [SMX] = 20 mg/L	100	-	Graphitic N	Electron transfer, O_2_^·−^	[55]
Lotus leaf	PDS	LLC800	Acid orange 7	T = 25 °C, PH = 6.4 ± 0.1, Catalyst = 0.25 g/L, [PDS]= 4 g/L, [AO7] = 200 mg/L	99.46	N.R	Biochar Surface	SO_4_^·−^, ·OH, ^1^O_2_, O_2_^·−^	[57]
Bean dreg	PDS	BDK900	Bisphenol A	Catalyst = 0.1 g/L, [PDS]= 5 mM, [BPA] = 80 mg/L	100	0.4296	Pyridinic N	Surface-bound radical, Electron transfer	[58]
Rice straw	PMS	NRSBC800	Acid orange 7	T = 25 °C, Catalyst = 100 mg/L, [PMS]= 2 mM, [AO7] = 50 mg/L	100	0.21	Graphitic N, Pyridinic N, Pyrrolic N	SO_4_^·−^, ·OH, ^1^O_2_, O_2_^·−^	[64]
Straw	PDS	N-BC	Tetracycline	T = 25 °C, Catalyst = 200 mg/L, [PDS]= 2 mM, [TC] = 20 mg/L	100	-	Graphitic N, Defect edge, Graphitization structure	Surface-bound reactive species, Electron transfer	[68]
Sorghum stalk	PDS	SG650	Sulfadiazine	T = 25 °C, pH = 5.8, Catalyst = 1.8 g/L, [PDS]= 9.1 mM, [SDZ] = 36.3 μM	94.4	0.0102	PFR, Sp^2^-C	Electron transfer, ^1^O_2_	[86]
Reed	PDS	N-BC	Orange G	T = 25 °C, pH = 5.8, Catalyst = 0.2 g/L, [PDS]= 2 mM, [OG] = 50 ppm	100	0.039	C=O, Defect sites, N-doped sites, Sp^2^-C	Electron transfer, ^1^O_2_	[63]
Wood residue	PMS	NC800–20	Acid orange 7	T = 25 °C, pH = 3–4, Catalyst = 0.1 g/L, [AO7] = 10 mg/L, AO7:PMS ratio = 1:50	100	0.342	Graphitic N, C=O, Pyridinic N, Pyrrolic N	SO_4_^·−^, ·OH, ^1^O_2_, Electron transfer	[60]
Sludge	PMS	NSDB800	Sulfamethoxazole	T = 25 °C, pH = 3–4, Catalyst = 0.2 g/L, [SMX] = 0.04 mM, [PMS] = 0.8 mM	100	-	Grapitic N	Surface-bound reactive species	[67]
Spent coffee ground	PMS	PC-SC	Bisphenol A	T = 25 °C, pH = 4, Catalyst = 0.2 g/L, [BPA] = 5 mg/L, [PMS] = 0.3 g/L	95%	0.072	Graphitic N, Sp^2^-C	^1^O_2_	[52]
Sawdust	PMS	N-C-d-4–800	Bisphenol A	T = 25 °C, pH = 6.28, Catalyst = 0.5 g/L, [BPA] = 10 mg/L, [PMS] = 2 mM	100%	1.48	Graphitic N, Pyridinic N, Defect sites	SO_4_^·−^, ·OH, ^1^O_2_, Electron transfer	[54]

However, some studies believe that the incorporation of N atoms into biochar will weaken the effect of C=O in the activation of PMS to produce ^1^O_2_ [61]. Wang et al. [31] proposed an electron transfer pathway involving surface-bound reactive complexes for the degradation of sulfadiazine (SDZ) by activating PDS using NBCs prepared from corncob biomasses and urea (Equations (9)–(11)). The PS-AOP system with the electron transfer pathway as the main pathway not only possesses broad pH adaptability, but also exhibits high resistance to inorganic anions in the aquatic environment.

(9) NBC+PDS→[NBC−PDS]

(10) $$[\overset{}{\overset{︷}{NBC-P}}\vec{e}DS]\to NBC_{OX}+2SO_{4}^{2-}$$

(11) $$\overset{}{\overset{︷}{SDZ+[N}}\vec{e}\overset{}{\overset{︷}{BC-P}}\vec{e}DS]\to SDZ_{OX}+NBC+2SO_{4}^{2-}$$

Since non-radical pathways mainly occur at the NBC surface, the higher adsorption capacity and similar adsorption rates allow more targeted organics to participate in the charge-transport process. Therefore, the enhanced adsorption between organics and NBCs determines the non-radical oxidation rate.

**Figure 3:**
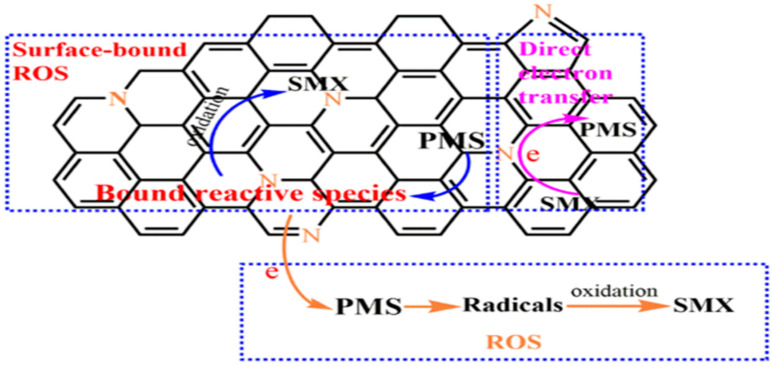
Proposed mechanism of PMS catalytic process by N-doped sludge-derived biochar [67], copyright 2021, Elsevier.

Another pathway is to complete the electron transfer with PMS through NBCs to generate free radicals, but it is not equivalent to direct electron transfer pathway. Wang et al. [67] proposed that PMS was adsorbed on the NBC surface to produce the surface-bound reactive species by inner-sphere complexation, then the reactive species reacted with sulfamethoxazole (SMX) resulting in the SMX degradation, as shown in Figure 3.

In summary, it can be concluded that the current pathway for PDS/PMS activation by NBC is dominated by ^1^O_2_, electron transfer and surface-bound radicals. It is supplemented by SO_4_**^·^**^−^ and ·OH, both of them have great effects on the degradation of organic pollutants. Besides the above mentioned C=O, the graphitization degree of NBC, the degree of defects, especially the type of N configurations, are all affect the catalytic activity of NBC to varying extents. The related discussions will be presented in Section 4.1 and Section 4.2.

### 3.2. Modified NBC for PS-AOP

**Figure 4:**
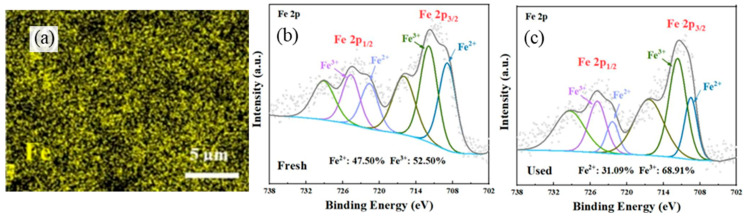
(**a**) The Fe element mapping of ISBC; (**b**) Fe 2p XPS spectra of fresh ISBC; (**c**) Fe 2p XPS spectra of ISBC used for 4 cycles in ISBC/PMS system [74], copyright 2022, Elsevier.

Different from NBCs, the free-radical pathway in modified NBCs plays a more important role in the PS-AOP. Fu et al. [74] prepared iron species self-doped biochar derived from municipal sludge by a simple method of EDTA-citric acid leaching/pyrolysis, exploring an efficient PMS activation method for perfluorooctanoic acid (PFOA) degradation. As can be seen in Figure 4, element mapping was used to prove that there was a uniform distribution of iron on the surface of iron self-doped of sludge-derived biochar (ISBC), and the changes in Fe^2+^ and Fe^3+^ contents before and after catalysis confirmed that iron species induced free radicals to participate in PS-AOP as follows (Equations (12)–(15)).

(12) $$Fe^{2+}+HSO_{5}^{-}\to Fe^{3+}+SO_{4}^{•-}+OH^{-}$$

(13) $$Fe^{3+}+e^{-}\to Fe^{2+}$$

(14) $$SO_{4}^{•-}+H_{2}O\to SO_{4}^{2-}+HO^{•}+H^{+}$$

(15) $$Fe^{3+}+HSO_{5}^{-}\to Fe^{2+}+SO_{5}^{•-}+H^{+}$$

Similarly, the incorporation of S atoms into NBC forms thiophene S (C-S-C), which is also considered to facilitate the cleavage of O−O in PMS/PDS, and is a key active site for the generation of SO_4_**^·^**^−^ radicals [71,73,87]. However, the catalytic performance of a catalyst is not simply determined by the number of active sites. Ding et al. [61] prepared N@S co-doped biochar by rice straw for the catalytic degradation of metolachlor (MET) through activating PMS. It was found that N-doping positively whilst S-doping negatively influenced the MET degradation process. The S-doping modification results in negligible charge transfer between the involved C atoms and may disrupt the charge balance of the covalent carbon electron system, thereby disrupting charge redistribution. It should be noted that the synergistic effect largely depends on the preparation method, and the effect of the preparation method on the catalytic activity needs to be further studied.

**Figure 5:**
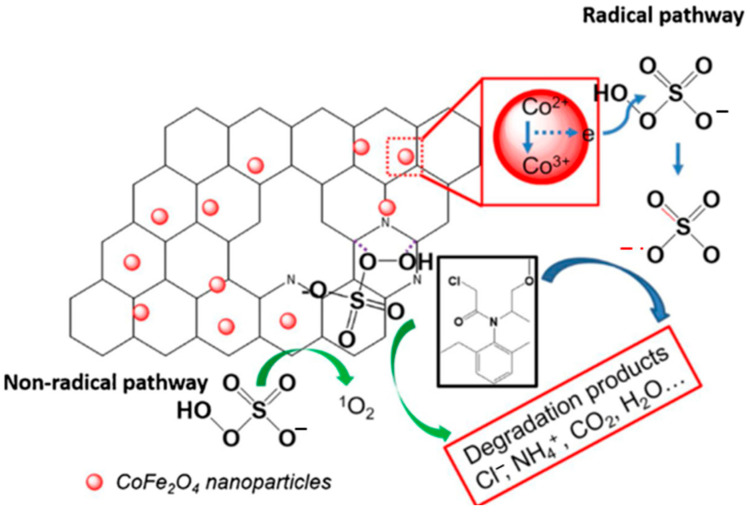
Mechanism of PMS activation by MNBC_800_ and MET degradation [34], copyright 2019, Elsevier.

Moreover, the use of NBC as a carrier to composite with spinel ferrites materials to achieve synergistic catalysis of the two materials has received more and more attention. Liu et al. [34] synthesized magnetic NBC-supported CoFe_2_O_4_ composite (MNBC) using agricultural waste straw as precursor. The prepared catalyst exhibited excellent performance in catalytic degradation of MET by coupling with PMS. As shown in the Figure 5, the CoFe_2_O_4_ nanoparticles supported on the surface of NBC are the active sites to generate sulfate through the redox reaction of Co^2+^ and PMS (Equations (16)–(18)) [88,89].

(16) $$Co^{2+}+HSO_{5}^{-}\to Co^{3+}+SO_{4}^{•-}+OH^{-}$$

(17) $$Co^{3+}+HSO_{5}^{-}\to Co^{2+}+SO_{5}^{•-}+H^{+}$$

(18) HSO5−+SO52−→SO42−+HSO4−+O21

Table 3 shows some emerging materials of modified NBC materials as PS-AOP heterogeneous catalysts. It can be found that the Fe is most commonly used in the manufacture of modified NBCs. In addition, modified NBCs have high-activation properties for PMS/PDS; therefore, these studies provide directions for the development of other high-performance and stable NBC materials for environmental remediation.

### 3.3. Detection Technologies for Activation Mechanism

At present, chemical quenching experiments and electron spin resonance (ESR) are adopted to detect the ROS of NBCs in PS-AOP. For the free radicals that widely exist in the NBC/PS system, the role of the corresponding free radicals in the degradation process can be analyzed by adding a quencher to conduct a comparative experiment. Currently, the quenchers used in the NBC/PS chemical quenching experiment mainly include ethanol (EtOH), methanol (MEOH), Tert-butanol (TBA), P-Benzoquinone (PBQ), lycopene (LCP), Nitrobenzene (NB), and phenol. Table 4 lists the second-order reaction constants of commonly used quenchers to ROS. ETOH and MeOH are usually used as quenchers for ·OH and SO_4_^·−^ due to their high reaction rates with ·OH and SO_4_^·−^. TBA has a much lower reaction rate for SO_4_^·−^ than ·OH; therefore, it is used to quench ·OH. LCP and PBQ are used as quenchers for ^1^O_2_ and O_2_^·−^, respectively. In addition, for surface-bounded radicals, Ye et al. [85] used a hydrophobic radical quencher to analyze the degradation pathway by selectively terminating the surface free-radical reaction.

**Figure 6:**
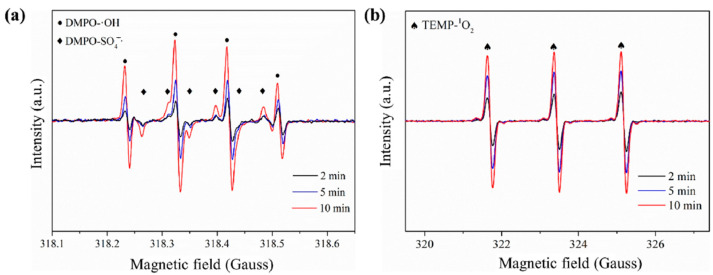
Detected ESR signals by using (**a**) DMPO and (**b**) TEMP as the spin trapping agents [56], copyright 2020, Elsevier.

In addition to chemical quenching experiments, ESR is also often used to further identify the type of generated free radicals. The spin-trapping method is to add an unsaturated anti-magnetic compound (spin traps) into the reaction system, and the combination of free radicals and spin traps forms a relatively stable spin adduct [70]. 2,2,6,6-Tetramethyl-4-piperidinol (TEMP) and 5,5-dimethyl-1-pyrrolidine N-oxide (DMPO) are commonly used as spin traps. As shown in Figure 6, the ESR signals with hyperfine coupling constants of α_H_ = 1.44 G, α_H_ = 0.76 G, α_N_ = 15.02 G, and α_H_ = 14.81 G were assigned to be the DMPO-SO_4_ adduct. The ESR signals with hyperfine coupling constants of α_N_ = 15.05 G and α_H_ = 14.21 G were attributed to the DMPO-OH adduct. The triplet ESR signal with the same intensity ratio (1:1:1, α = 17.2 G) was corresponded to the oxidized TEMP by ^1^O_2_ [34].

**Figure 7:**
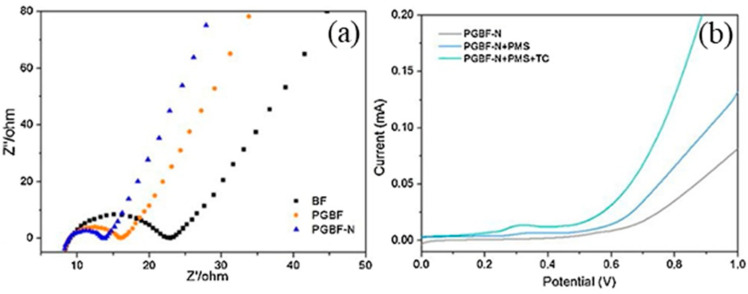
(**a**) EIS measurement of biochar-based catalysts and (**b**) LSV under different conditions [85], copyright 2020, Elsevier.

Electrochemical experiments are generally used to explore the non-radical pathways of NBC/PS. In the electrochemical impedance spectroscopy (EIS) Nyquist plot, the diameter of the semicircle presented by the NBC is proportional to its charge-transfer resistance [63,96,97]. Linear sweep voltammetry (LSV) is to further explore the electron transfer process, and the strong current response indicated NBCs have good electrical conductivity [86,98]. Ye et al. [85] studied the electron transfer process by LSV and EIS. Through the comparison of EIS, they found that the incorporation of N and the increase in the graphitization degree made the graphitic biochar fiber doped with N (PGBF-N) have lower impedance and stronger electron transfer abilities, as shown in Figure 7. Through the analysis of LSV, it was found that when using PGBF-N as the working electrode, the addition of PMS caused an increase in the current, which implies the interaction and electronic rearrangement between the PMS and the PGBF-N. The addition of contaminants leads to another current enhancement, demonstrating fast electron transfer over the established PMS/PGBF-N/Tetracycline ternary system, where the current forms a bridge across PBGF-N to facilitate the transfer of electrons from TC molecules to metastable PMS.

**Figure 8:**
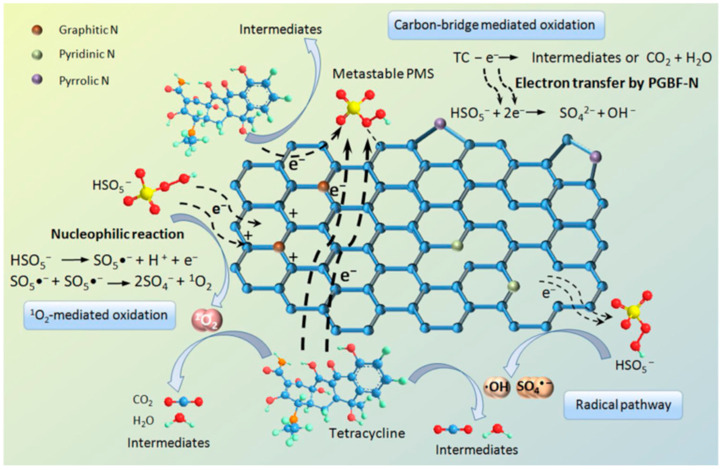
Proposed mechanism of PMS activation by PGBF-N for the degradation behavior of TC molecules [85], copyright 2020, Elsevier.

Figure 8 details the PMS activation mechanism of PGBF-N on the degradation behavior of the above TC molecules. The PMS molecule is gaining electrons to generate free radicals. These things considered, the positive charge on the adjacent carbon of the graphitic N induces the PMS molecule to lose electrons to generate ^1^O_2_ through a nucleophilic reaction. A direct electron-transfer pathway also exists, since the addition of Sp^2^-C promotes graphitization to a degree that shows better electrical conductivity than sp^3^-hybridized carbons (Sp^3^-C). Similar to the degree of graphitization, the N configuration of NBC also has a great influence on the catalytic activity, which will be further explained in the next chapter.

## 4. Structures of NBCs Affecting the PS Activation and the Role of N Configuration

### 4.1. Structures of NBCs Affecting the PS Activation

As above discussed, the catalytic activity of biochar is closely related to its adsorption capacity, charge transfer capacity, and potential active sites. Therefore, we could regulate the structures of NBC including SSA, defect degree, and graphitization degree to promote its catalytic activity.

**Figure 9:**
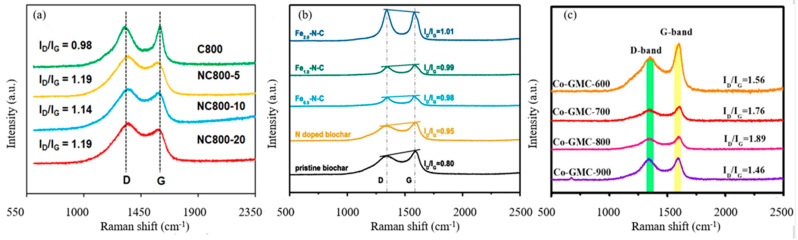
(**a**) Raman spectra of various catalysts [60], copyright 2020, Elsevier; (**b**) Raman spectra of pristine biochar, NBC and Fe/N co-doped biochar materials [54], copyright 2020, Elsevier; (**c**) Raman spectra of as-prepared composites [100], copyright 2020, Elsevier.

The carbon structure of NBCs can be studied by Raman spectra. The D band (~1350 cm^−1^) is the result of disordered levels caused by vacancies, zigzag/armchair edges, functional groups, and heteroatom doping. The G band (~1580 cm^−1^) is related to the E_2_g mode vibration of sp^2^- hybridized carbon domains [99]. The ratio of I_D_/I_G_ reveals the defect degree and graphitization degree of NBCs. Zaeni et al. [60] compared the degree of structural defects of pristine biochar and NBCs, and the I_D_/I_G_ value of NBCs was higher than that of BC, as shown in Figure 9a. One commonly accepted theory at the moment is that N-doping increases the distortion of carbon layers and creates more defect sites [31,61,63]. These defect sites are beneficial because they can perturb the electronic charge distribution of the conjugated carbon system and act as redox-active functional groups for PMS activation. Xu et al. [54] compared the defect degree of pristine biochar, NBCs, and Fe@N co-doped biochar as shown in Figure 9b, and the Fe@N co-doped biochar was found to exhibit a higher degree of defects. The results indicated that the co-doping of iron and N would lead to the distortion of the carbon network and generate more defects. Abundant defects due to zigzag/armchair edges, vacancies, and functional groups in carbon-based catalysts help to promote the adsorption and activation of PMS [42]. Besides heteroatom doping, pyrolysis temperature also is an important factor affecting I_D_/I_G_. As shown in Figure 9c, Luo et al. [100] compared biochars prepared at different temperatures and found that the I_D_/I_G_ ratio increases with the increase in the pyrolysis temperature (<800 °C), indicating more defects were formed; however, when the temperature reached 800 °C, the I_D_/I_G_ decreases significantly, indicating that a higher degree of graphitization is obtained. Studies have shown that the graphitic structure contributes to the charge-transfer process, and the high degree of graphitization helps to facilitate electron transfer between PMS and carbon catalysts, thereby promoting non-radical degradation pathways [32]. Moreover, this study also reported that a high degree of graphitization promotes charge transfers to enhance PS activation, while graphitized carbon structures with many defects can also promote a charge transfer and lead to non-radical pathways. Therefore, both the high-defect degree and the high-graphitization degree can promote PS activation at different levels.

The SSA and pore structure of NBCs are another key factor affecting its catalytic activity in PS-AOP. Zhu et al. [63] reported that N-doping enhanced the specific surface area of NBCs derived at 900 °C (496.7 m^2^g^−1^) compared to that of primary biochar derived at 900 °C (95.2 m^2^g^−1^) due to the N precursors also releasing gases that further adjust the porous structure of the biochar. In addition, for NBCs, at a high-pyrolysis temperature above 750 °C, the SSA will greatly increase due to the volatilization of tar compounds, thus forming more porous structures [101]. The successful performance of NBC in the catalytic oxidation of organic pollutants can partially attributed to a large SSA which provides more reactive site [102]. Wang et al. [31] also reported that apparent rate constant (k) and SSA have a close correlation and that the correlation coefficient is 0.981. However, porosity is inversely proportional to the graphitization degree, hence the NBC needs an appropriate balance between mass transfer and conductivity [85]. Appropriate SSAs and pore distribution are conducive to exposing more reaction sites for easy contact of the catalysis with substrates without damaging electron conduction [85,102].

### 4.2. The Role of N Configuration in PS Activation

N-doping is one of the simplest and most promising methods to enhance the reactivity of catalysts; N-doping with localized unpaired electrons is capable of (1) increasing the electron density of adjacent carbon atoms, (2) the electron flow in Sp^2^-C is enhanced by conjugation [42], (3) producing more functional groups and defects, and (4) increasing the surface polarity of carbon materials and attracting polar adsorbents. However, as for the reaction sites of PS-AOP, it is still controversial whether the reaction sites are caused by pyridinic N, pyrrolic N, or graphitic N [103]. Oh et al. [33] found that NBC prepared at 1000 °C is rich in graphitic N, which acting as a possible active site for ^1^O_2_ generation through non-radical pathway. Meanwhile, pyrrolic N and pyridinic N are conducive to redox reaction and vital for radical pathway. This is similar to the rule of the degradation pathway corresponding to the active sites listed in Table 2.

On this basis, Hu et al. [59] found that graphitic N can accelerate the electron transfer between adjacent carbon atoms and destroy the inertia of conjugated graphitized carbon networks, which will increase the positive charge of carbon atoms. It is favorable to weaken the O-O bond and form surface metastable PMS through electron rearrangement or generate reactive substances by nucleophilic addition reaction of the PMS towards positively charged carbon (Equations (19) and (20)) [104]. Furthermore, the pyridinic N with the long-pair electrons could promote the transfer of free-flowing π-electrons from the Sp^2^-C of biochar to activate PMS and further generated SO_4_**^·^**^−^ and ·OH [105]. In addition, pyrrolic N could adsorb pollutant molecules, which could accelerate the formation of complexes and boost the transfer of electrons [77].

(19) $$HSO_{5}^{-}\to SO_{5}^{•-}+H^{+}+e$$

(20) SO5•−+SO5•−→O21+2SO4−

**Figure 10:**
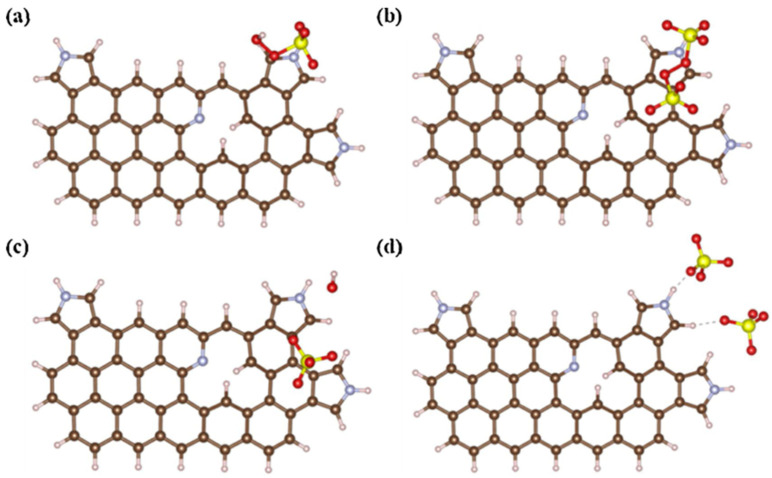
Adsorption of (**a**) PMS (**b**) PDS and the corresponding desorption of (**c**) PMS and (**d**) PDS on pyrrolic N [58], copyright 2021, Elsevier.

However, some studies put forward different views. Wang et al. [31] found that the incorporation of edge N configuration (pyridinic N and pyrrolic N rather than graphitic N) generate reactive sites for the PDS activation, and a non-radical pathway (electron transfer) involving surface-bond reactive complexes was proved to play a major role in the NBC/PDS system. For the research on the important role of non-graphitic N in the non-radical pathway, it was assumed that the type of persulfate plays an important role. Cai et al. [58] analyzed the adsorption behaviors of PDS and PMS on pyrrolic N as shown in Figure 10. The adsorption energy and dissociation adsorption energy of PDS (−0.24 and −2.71 eV) on pyrrolic N were higher than PMS (−1.10 and −0.78 eV), which indicated that pyrrolic N-rich biochars exhibited better adsorption towards PDS than PMS.

To sum up, graphitic N is the dominant N configuration of NBCs in the non-free-radical pathway of PS-AOP, while pyridinic N and pyrrolic N play more important role in the free-radical pathway. The type of PS is another key factor determining the participation degree of pyridinic N and pyrrolic N in the non-free-radical pathway.

## 5. Conclusions and Outlook

In conclusion, we comprehensively reviewed the research progress of NBCs in PS-AOP to treat organic pollutants in water. The in situ N-doped method and the post-treatment method are most commonly used to prepare NBC materials. Aquatic plants are suitable for the in situ N-doped method, while sludge and bamboo are suitable for the post-treatment method. In addition, biomasses with high inorganic salt content or leaf veins are not suitable for NBC preparation. The addition of other atoms in NBCs will induce the generation of free radicals through charge transfers and the change in the valence state of metal ions, so that the original non-free radical-dominated degradation pathway will be transformed into free-radical-dominated degradation pathway. As a heterogeneous catalyst for PDS/PMS, the above NBC shows excellent catalytic performance in removing organic pollutants. A variety of methods can be used to determine the activation mechanism, including chemical quenching experiment, ESR detections, EIS detections, and LSV detections.

Moreover, we further summarized the influence of various physical and chemical properties of NBCs on the catalytic capacity. The degree of graphitization and the degree of defect cooperatively promote the activation of PS, and the opposite relationship between the SSA and the degree of graphitization requires NBCs to strike a proper balance between mass transfer and electrical conductivity. Graphitic N is considered to be the dominant N configuration of non-radical pathways, while pyridinic N and pyrrolic N play more important roles in radical pathways. The type of PS is one of the key factors affecting the dominant N configuration of NBC in PS-AOP.

However, the current studies are mainly focused on fundamental research at the laboratory scale, and commercial applications that treat real wastewater are insufficient. Moreover, there are only a few papers on the reuse performance of NBC catalysts, although it is a significant property for a catalyst. In addition, I_D_/I_G_ values obtained from Raman analysis combined with XRD analysis are commonly used to characterize both the graphitization degree and defect degree of NBCs, which is not precise enough. It is highly demanded to find a more precise technology, not only for the NBCs but also for all biochars and even carbon materials. Last but not least, the treatment of by-products from producing NBCs, e.g., bio-oil and toxic gases, needs to be considered for both the economic and environmental benefits.

For further investigations, the following recommendations are given. First, it is necessary to develop a method that precisely regulates the structure of NBCs, especially its N configuration, to improve the catalytic performance. Currently, there is no preparation method that can design the N configuration or the content of the targeted type of N functional group. Second, the degradation mechanism and catalytic effect of the same NBC on different organic compounds are different. The relationship between the catalytic performance of NBCs and the structure of degraded organic compounds should be established. This helps to determine the structure of NBCs that achieve the best catalytic activity for the targeted organic compound. Third, systematic research should be conducted on the differences in selectivity, oxidation potential, and degradation pathways between free radicals and non-free radicals.

## Figures and Tables

**Table 3:** List of modified NBC materials as catalysts for PS-AOP.

Biomass	Oxidant	Attached FunctionalGroups	Catalysts	Pollutant	Reaction Conditions	Removal Efficiency (%)	Rate Constant (min^−1^)	Active Sites	Activation Mechanism	Ref.
Rice straw	PMS	CoFe_2_O_4_	MNBC800	Metolachlor	pH = unadjusted, Catalyst = 0.2g/L, [MET] = 10 mg/L, [PMS] = 0.5 mM	100	0.104	Graphitic N, Co^2+^	SO_4_^·−^, ·OH, ^1^O_2_, Electron transfer	[34]
Sludge	PMS	Co, S	Co_9_S_8_@N-S-BC	Sulfamethoxazole	T = 25 °C, pH = 3, Catalyst = 0.2 g/L, [NOR] = 10 mg/L, [PMS] = 1.6 mM	100	0.379	Carbon defects, Quaternary N, the carbon atoms next to pyridinic N, C=O, -C-S-C-, Co (II)	SO_4_^·−^, ·OH	[90]
Maize straw	PDS	Fe	Fe@N co-doped biochar	Norfloxacin	T = 25 °C, pH = 7, Catalyst = 0.1 g/L, [SMX] = 0.08 mM, [PMS] = 10 mmol/L	96.45	0.258	Fe, Graphitic N, C-OH/C = N	SO_4_^·−^,· ·OH, ^1^O_2_	[79]
Banyan	PMS	Fe, Ce	Fe-Ce@N-BC	Metronidazole	T = 25 °C, pH = 5.74, Catalyst = 0.75 g/L, [MNZ] = 0.01 g/L, [PMS] = 2 mM	97.5	0.0566	Graphitic N, Pyridinic N, C=O, Defects, Fe^2+^/Fe^3+^, Ce^3+^/Ce^4+^	SO_4_^·−^, ·OH, ^1^O_2_	[91]
Sawdust	PMS	Fe	Fe-N-C-BPA	Bisphenol A	T = 25 °C, pH = 6.76, Catalyst = 0.1 g/L, [BPA] = 0.01 g/L, [PMS] = 0.5 mM	97	0.0556	Fe-Nx, Pyridinic N, graphitic N, Fe_2_O_3_, Fe^0^	SO_4_^·−^ ·OH, ^1^O_2_	[78]
Rice husk	PMS	Fe_3_O_4_, NCNT	Fe_3_O_4_@NCNTs-BC800	Sulfamethoxazole	T = 25 °C, Ph = 7, Catalyst = 0.4 g/L, [SMX] = 0.01 g/L, [PMS] = 0.6 mM	98.2	0.092	Pyridinic N, Fe (II), Fe (III)	Surface bound O_2_^·−^, ·OH, SO_4_^·−^, Electron transfer	[77]
Human hair	PMS	S	NSC-800	Bisphenol A	Catalyst = 0.08 g/L, [BPA] = 25 mg/L, [PMS] = 0.4 g/L	98.4	-	Graphitic N, Sp^2^-C, -C-S-C, Defect sites	^1^O_2_, ·OH, SO_4_^·−^	[87]
Glucose	PDS	Cu	N-Cu-biochar	Tetracycline	Catalyst = 200 mg/L, pH = 5, [TC] = 20 mg/L, [PDS] = 2 mM	100	0.0482	Cu^2+^	·OH, SO_4_^·−^, Electron transfer	[75]
Maso bamboo	PMS	S	NSBC-500	Antibiotic	Catalyst = 3 mg/L, [antibiotic] = 20 mg/L, [PMS] = 5 mM	70.97	0.0274	EPFR, Defect structure	SO_4_^·−^, ·OH, ^1^O_2_, O_2_^·−^	[51]
Camphor sulfonic	PDS	S	NSC-750	Sulfamethoxazole	pH = 5, Catalyst = 0.2 g/L, [SMX] = 20 mg/L, [PDS] = 0.4 mM	96	0.0348	Pyridinic N, C-S-C, Defect sites, C=O	^1^O_2_, ·OH, SO_4_^·−^, Electron transfer	[71]
Sludge	PDS	Fe	MS-800	Tetracycline	pH =2.17, Catalyst = 0.2 g/L, [TC] = 100 mg/L, [PDS] = 4.2 mM	82.24	0.0096	Fe species, Sp^2^-C, N species	·OH, SO_4_^·−^	[92]
Wheat straw	PDS	Fe	Fe-N-BC	Acid orange 7	pH = 3, Catalyst = 0.2 g/L, [AO7] = 20 mg/L, [PDS] = 1 mM	100	0.114	Fe species, N species, PFR	^1^O_2_, SO_4_^·−^, ·OH, O_2_^·−^, Surface-bounded radical, Electron transfer	[80]
Wood chip	PDS	Fe, K	KMBC	Metronidazole	T = 25 °C, pH = 6.5, Catalyst = 0.5 g/L, [MNZ] = 20 mg/L, [PDS] = 1 mM	98.4	0.025	Fe(II) PFR	^1^O_2_, SO_4_^·−^, ·OH, O_2_^·−^, Surface-bounded radicals, Electron transfer	[93]
Banana	PDS	Fe_2_O_3_	Fe_2_O_3_@BC-2	Bisphenol A	T = 25 °C, pH = unadjustment, Catalyst = 0.3g/L, [BPA] = 20 mg/L, [PDS] = 5 mM	100	0.1849	Pyridinic N, Graphitic N, -OOH, -OH Defect sites, PFR, Fe species	SO_4_^·−^, ·OH, O_2_^·−^	[94]
Melamine	PDS	S	ACO850-20N20S	Methyl orange	T = 30 °C, pH = 5, Catalyst = 0.8 g/L, [MO] = 200 mg/L, [PDS] = 1.2 g/L	99	0.0075	C=O, C-S-C, Graphitic N, Pyridinic N	Surface bound radical	[73]
Sludge	PDS	Fe	SDBC	Sulfamethoxazole	T = 25 °C, pH = 5, Catalyst = 2.0 g/L, [SMX]= 40 μM, [PDS] = 1.5 mM	94.6	0.0145	Fe species, N species	^1^O_2_	[49]
Sludge	PMS	Fe	ISBC	Perfluorooctanoic acid	T = 60 °C, pH = 6.4, Catalyst =1 g/L, [PFOA] = 2 mg/L, [PMS] = 10 mM	99.9	0.054	Pyridinic N, C=O, Quinone groups	^1^O_2_	[74]

**Table 4:** The second-order reaction constants of commonly used quenchers to ROS.

Scavengers	Rate Constant (M^−1^s^−1^)				Ref.
	·OH	SO_4_^·−^	^1^O_2_	O_2_^·−^	
EtOH	1.2–2.8 × 10^9^	1.6–7.7 × 10^7^	-	-	[63,64]
MeOH	9.7 × 10^8^	3.2 × 10^6^	-	-	[68,75]
TBA	6 × 10^8^	4.0 × 10^5^	-	-	[56,79,92,94,95]
PBQ	-	-	-	9.6 × 10^8^	[62,77,79]
LCP	-	-	3.1 × 10^10^	-	[61]
Phenol	(surface) 8.8 × 10^9^	8.8 × 10^9^	-	-	[60,85]
NB	(surface) 3.9 × 10^9^	<10^6^	-	-	

## Data Availability

Data available within the article.
